# Herbivore and Fungal Pathogen Exclusion Affects the Seed Production of Four Common Grassland Species

**DOI:** 10.1371/journal.pone.0012022

**Published:** 2010-08-11

**Authors:** Timothy L. Dickson, Charles E. Mitchell

**Affiliations:** 1 Tyson Research Center, Washington University, St. Louis, Missouri, United States of America; 2 Biology Department, University of North Carolina, Chapel Hill, North Carolina, United States of America; University of Hull, United Kingdom

## Abstract

Insect herbivores and fungal pathogens can independently affect plant fitness, and may have interactive effects. However, few studies have experimentally quantified the joint effects of insects and fungal pathogens on seed production in non-agricultural populations. We examined the factorial effects of insect herbivore exclusion (via insecticide) and fungal pathogen exclusion (via fungicide) on the population-level seed production of four common graminoid species (*Andropogon gerardii*, *Schizachyrium scoparium*, *Poa pratensis*, and *Carex siccata*) over two growing seasons in Minnesota, USA. We detected no interactive effects of herbivores and pathogens on seed production. However, the seed production of all four species was affected by either insecticide or fungicide in at least one year of the study. Insecticide consistently doubled the seed production of the historically most common species in the North American tallgrass prairie, *A*. *gerardii* (big bluestem). This is the first report of insect removal increasing seed production in this species. Insecticide increased *A*. *gerardii* number of seeds per seed head in one year, and mass per seed in both years, suggesting that consumption of flowers and seed embryos contributed to the effect on seed production. One of the primary insect species consuming *A*. *gerardii* flowers and seed embryos was likely the Cecidomyiid midge, *Contarinia wattsi*. Effects on all other plant species varied among years. Herbivores and pathogens likely reduce the dispersal and colonization ability of plants when they reduce seed output. Therefore, impacts on seed production of competitive dominant species may help to explain their relatively poor colonization abilities. Reduced seed output by dominant graminoids may thereby promote coexistence with subdominant species through competition-colonization tradeoffs.

## Introduction

Seed production is an important component of fitness for most plants. Establishment from seed is particularly important for long-distance dispersal and the colonization of large-scale disturbances [1], [2]. Soil disturbance in the North American tallgrass prairie was historically small-scale and caused by large herbivores [3] and flooding, but large-scale disturbances currently exist in the form of agriculture, overgrazing, and human development. The local abundance of established plant populations can be directly limited by the production of viable seed [4], although limitation of seed production does not necessarily imply seed limitation of population abundance [5]. At larger spatial scales, lack of dispersed seed can limit the presence of species in communities where species would otherwise thrive [6]. Seed production may therefore limit plants' habitat occupancy as well as local abundance.

Pathogens and invertebrate herbivores are both ubiquitously associated with plants, and commonly co-occur or interact via shared plant hosts [7]–[10], and both can limit seed production in natural and agricultural landscapes. Pre-dispersal insect granivory typically removes between 10 and 90% of seeds in natural environments [11]–[13]. If crops were not treated with pesticides, pathogens and insects would cause estimated worldwide grain yield (i.e. seed production) losses equal to approximately one-third of attainable production [14]. In wild plant communities, pathogens can greatly reduce plant survival, growth, and seed set [15]–[19]. On the other hand, some studies, particularly ones that do not experimentally manipulate infection, find no negative effect of pathogens on seed production [18], [20], [21]. In at least one case, negative impacts of fungal pathogens on seed set were offset by positive impacts on germination [22], but the frequency of such positive effects is unknown.

Pathogens and invertebrate herbivores may decrease seed production both directly and indirectly. Directly, they can infect germ tissue and inhibit inflorescence production [23], or infect and kill seeds [24]. Indirectly, infection or consumption of vegetative tissue can decrease the resources available for allocation to seed production [5], [15]. For example, fungal pathogens can decrease photosynthetic capacity, leaf longevity, and root production of perennial grasses [25]. Further, invertebrate herbivores can act as keystone species, suppressing dominant species, and thereby indirectly increasing reproduction of subordinate species [26]. Notably, plants may compensate, at least partially, for negative impacts at both individual [8] and population [27] levels.

Together, these observations raise an important question: are the joint effects of pathogens and invertebrate herbivores on seed production, and plant performance more broadly, independent or interactive? In the last comprehensive review [28], the most common effect of combined pathogen-herbivore attack on plant performance was sub-additive, with additive effects being next most common, and synergistic and antagonistic effects being relatively rare. Hatcher [28] pointed out some important limitations of studies available at that time. Most studies were conducted on agricultural hosts. All wild hosts studied were grown in pots, and responses were largely measured at the individual plant level. Finally, most studies examined only a single plant species, and lasted less than a year. Together, these limitations mean that most published studies did not allow for some important processes that will influence long-term population responses, including: (A) effects requiring more than one season, including overwintering survivorship [7] and maternal effects [29]; (B) interannual variation in climate and other abiotic factors [28]; (C) compensation at the level of the plant population [27]; or (D) effects of pathogens or invertebrate herbivores mediated by interactions with other plant species [30]. A more recent meta-analysis [31] did not find a sufficient number of pathogen-herbivore studies to analyze separately, but its conclusions (and limitations) for enemy-enemy studies in general were consistent with those of Hatcher [28]. Since Hatcher's [28] review, a few field experiments have factorially manipulated pathogens and invertebrate herbivores in intact plant communities. These studies have found both additive [10], [25] and synergistic [10], [32] effects, but have examined only vegetative production, not seed production.

The goal of our study was to test for interactive effects of fungal pathogens and insect herbivores on seed production at the population level, in multiple plant species, and over multiple years. To achieve this, we factorially applied foliar insecticide and fungicide to naturally established grassland plots over 3.5 growing seasons. In the final two years, we quantified the response of the four most abundant plant species (*Andropogon gerardii*, *Schizachyrium scoparium*, *Poa pratensis*, and *Carex siccata*) in terms of seed production and vegetative abundance.

## Methods

The experiment was completed in existing vegetation in an old field at Cedar Creek Natural History Area, Bethel, MN, USA. Soils are sandy glacial outwash. The field was last cultivated in 1943, and has undergone natural succession since then. By the time of the study, the field was dominated by a native perennial grass, *A. gerardii*, with the second most abundant species being the non-native perennial grass, *P*. *pratensis* (see results for average species biomass). Another grass species (*S*. *scoparium*) and a sedge (*C*. *siccata*) also occurred in every plot. Each species was naturally infected by a pathogenic deuteromycete (asexual) fungus causing a leaf spot disease: *A*. *gerardii* by *Phyllosticta* sp.; *P*. *pratensis* by *Ascochyta* sp. (tentative); *S*. *scoparium* by *Colletotrichum* sp.; and *C*. *siccata* by *Septoria* sp. Additionally, *A*. *gerardii* was infected by the rust fungus *Puccinia andropogonis*.

Thirty-two plots, 2.5×2.5 m each and separated by 1 m walkways, were established in July 1996 in an area chosen to minimize plot-to-plot variation in vegetation. Insecticide (absence or addition) and fungicide (absence or addition) treatments were assigned to plots in a completely randomized factorial design, with eight replicates for each treatment combination. The non-systemic, broad-spectrum pesticides esfenvalerate (DuPont, USA) and mancozeb (Elf Atochem, Holland) were chosen as insecticide and fungicide, respectively. These pesticides minimize direct effects on non-target organisms (e.g. pollinators and mycorrhizae) while excluding target pathogens, herbivores, and granivores [25]. Specifically, mancozeb remains in the top 2.5 cm of the soil and breaks down with a half-life of four days or less [33], and mancozeb appears to have minimal influence on arbuscular mycorrhizae colonization [25], although it is possible that mancozeb affects mycorrhizal composition. Esfenvalerate contains essentially no nutrients, whereas the addition of mancozeb added about 0.50 g N m^−2^ year^−1^. A greenhouse study in the near-absence of insects and fungal pathogens found no significant effects of esfenvalerate or mancozeb on the growth of *A*. *gerardii*, *S*. *scoparium*, or *P*. *pratensis*, indicating that the pesticides do not directly affect plant growth [25]. A similar greenhouse test also found no direct effects of mancozeb on 18 wild clover species from California [34]. Following the establishment of plots in July 1996, insecticide and fungicide were sprayed every 7–10 days through 1999. Plots were sprayed during the growing season, generally May to September. Sprays were directed uniformly across all aboveground vegetation, including both leaves and seed heads, when present. Fungicide reduced the percentage of leaf area infected from 1–9% to nearly 0%, while insecticide reduced leaf herbivory non-significantly, likely because leaf herbivory was well less than 1% even in the absence or insecticide [25].

We measured seed production in 1998 and 1999 from the four most common species (listed above), which together composed 95% of total above-ground biomass harvested in 1999 [25]. In each plot, we counted the number of seed heads of each species and then randomly chose 25% of the seed heads from which to collect seed (up to 10 seed heads per plot). Seeds were collected by placing a mesh bag over each seed head shortly before it began to drop seeds. We removed bagged seed heads after all seeds were mature, removed all seeds from the seed head without removing the seed bristles, dried the seeds, and weighed the seeds. Some bagged seed heads were lost, resulting in no seed heads collected from some plots. The exact numbers of seed heads collected from each plot are listed in Dataset S1. Total seed mass per plot was calculated by multiplying the average mass of seeds per seed head harvested within the plot by the total number of seed heads within the plot. To gain a better understanding of the effects of treatments on the most dominant species, we also counted the number of seeds per seed head for *A*. *gerardii* and calculated the average mass per seed.

We quantified peak aboveground plant biomass of the four study species in mid-August 1999 by clipping two strips per plot, each 1.5 m×0.1 m. We sorted this biomass to species, dried it, and weighed the biomass. We also estimated the percent coverage of each species in two 1 m×0.5 m quadrats in July of each year, and we use these data to calculate species richness values.

### Analyses

All statistical analyses, except power analyses, were completed in SAS for Windows 9.1.3, Service Pack 4. To test the effects of treatments on total seed mass per square meter in 1998 and 1999, we initially performed repeated-measures Analysis of Variance (RM-ANOVA) on the data for each species. When a treatment x year interaction occurred, we used the “slice” function in SAS to examine whether the treatment had a non-significant effect in one year [35]. We refer to the slice function as a contrast in the results section because the slice function is similar to a contrast statement, except the slice function uses the pooled error term over both years. Specifically, if the variability among replicates (plots) within each year is low then the degrees of freedom will approximately equal the number of replicates summed over all years, whereas if the variability among replicates within each year is high then the degrees of freedom will approximately equal the number of replicates per year (P.M. Dixon, personal communication). In the years where treatments significantly affected total seed mass, we conducted further analyses to determine the effects of treatments on the number of seed heads per square meter and the mass of seeds per seed head (total seed mass per square meter  =  number of seed heads per square meter * mass of seeds per seed head). For the dominant species in the experiment, *A*. *gerardii*, we further examined the effects of treatments on the number of seeds per seed head and the mass per seed (mass of seed per seed head  =  number of seeds per seed head * mass per seed). When reading the results it is important to remember that a 44% average increase in number of seed heads per square meter and a 30% average increase in the mass of seeds per seed head will not necessarily lead to an 87% average increase in the total seed mass per square meter due to covariation between the number of seed heads per square meter and the mass of seeds per seed head.

We also examined the statistical power (1-β) of the insecticide x fungicide interactions using G*Power Version 3.1.2. We calculated post-hoc achieved power for ANOVA interactions and used inputs of 0.05 for alpha, 32 for total sample size, 1 for the numerator degrees of freedom, and 4 for the number of groups. We calculated power for “medium” effect size (f = 0.25) and “large” effect size (f = 0.40) [36] and found power equal to 0.277 and 0.589, respectively.

All total seed mass per square meter data were square root transformed to improve homoscedasticity, and error bars in the figures were back-transformed. No other data were transformed. All raw data are shown in Dataset S1. We used a level of significance of P<0.05.

## Results

**Figure 1:**
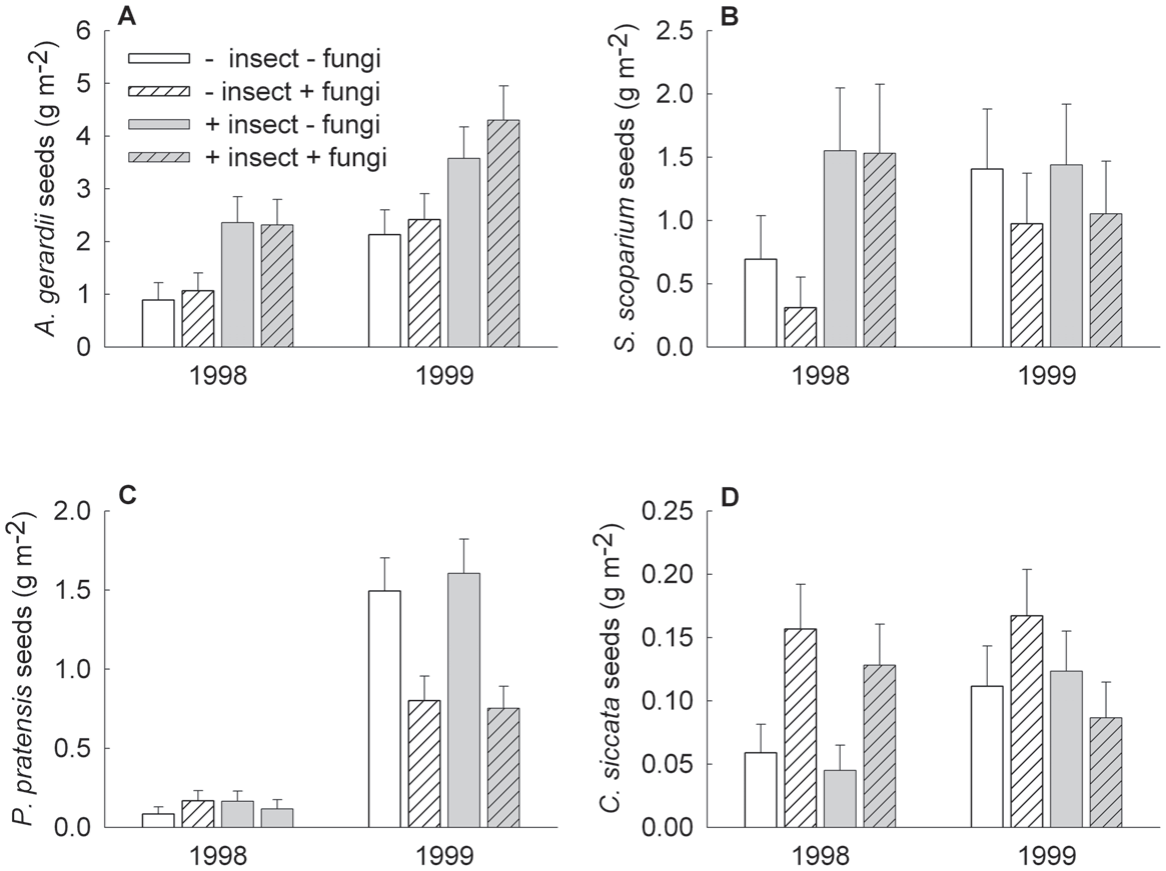
The effects of insecticide, fungicide, and year on total seed mass per square meter. Effects are shown for (A) *Andropogon gerardii*, (B) *Schizachyrium scoparium*, (C) *Poa pratensis*, and (D) *Carex siccata* (error bars are +1 standard error).

*Andropogon gerardii* seed mass per square meter was greater in 1999 than 1998, and insecticide increased *A*. *gerardii* seed mass per square meter by 101% when averaging across both years (Table 1, Fig. 1A). Insecticide increased the number of *A*. *gerardii* seed heads per square meter 98% (from 6.5 to 12.9) in 1998 and 26% (from 17.2 to 21.8) in 1999 (insecticide: F_1, 28_ = 6.36, P = 0.018; year: F_1, 28_ = 55.35, P<0.001), and increased the mass of each *A*. *gerardii* seed head 30% (from 0.14 to 0.18 g) averaged across both years (insecticide: F_1, 28.3_ = 37.41, P<0.001). No other predictor variable or interaction significantly affected *A*. *gerardii* number of seed heads per square meter or average mass per seed head.

**Table 1:** The effects of insecticide, fungicide, and year on total seed production for the four graminoid species (+ positive main effect of treatment; - negative main effect of treatment).

	Andropogon gerardii		Schizachyrium scoparium		Poa pratensis		Carex siccata	
Insecticide (I)	F_1, 27.5_ = 14.85 +	P<0.001	F_1, 28.1_ = 1.63	P = 0.211	F_1, 24.7_ = 0.23	P = 0.635	F_1, 27.1_ = 1.27	P = 0.270
Fungicide (F)	F_1, 27.5_ = 0.18	P = 0.675	F_1, 28.1_ = 2.07	P = 0.161	F_1, 24.7_ = 7.85 -	P = 0.010	F_1, 27.1_ = 3.91	P = 0.058
I x F	F_1, 27.5_ = 0.00	P = 0.948	F_1, 28.1_ = 0.06	P = 0.811	F_1, 24.7_ = 1.64	P = 0.212	F_1, 27.1_ = 1.27	P = 0.270
Year (Y)	F_1, 26.9_ = 43.05	P<0.001	F_1, 26.8_ = 5.10	P = 0.032	F_1, 21.6_ = 216.57	P<0.001	F_1, 25.9_ = 6.18	P = 0.020
Y x I	F_1, 26.9_ = 0.01	P = 0.904	F_1, 26.8_ = 5.18	P = 0.031	F_1, 21.6_ = 0.00	P = 0.985	F_1, 25.9_ = 0.25	P = 0.622
Y x F	F_1, 26.9_ = 0.70	P = 0.411	F_1, 26.8_ = 0.01	P = 0.922	F_1, 21.6_ = 17.33	P<0.001	F_1, 25.9_ = 9.75	P = 0.004
Y x I x F	F_1, 26.9_ = 0.87	P = 0.360	F_1, 26.8_ = 0.03	P = 0.872	F_1, 21.6_ = 0.11	P = 0.741	F_1, 25.9_ = 2.71	P = 0.112

Insecticide increased the seed mass per square meter of another C_4_ grass, *Schizachyrium scoparium*, by 218% in 1998 (Table 1, Fig. 1B), but had no significant effect in 1999 (1999 insecticide contrast: F_1, 38.9_ = 0.03, P = 0.866). In 1998, insecticide increased the number of *S*. *scoparium* seed heads per square meter 119%, from 6.4 to 14.0 (1998 insecticide: F_1, 28_ = 4.61, P = 0.041), and had no significant effect on the average mass of each *S*. *scoparium* seed head. No other predictor variable or interaction significantly affected *S*. *scoparium* number of seed heads per square meter or average mass per seed head in 1998.

Fungicide decreased *Poa pratensis* seed mass per square meter by 50% in 1999 (Table 1, Fig. 1C), but had no significant effect in 1998 (1998 fungicide contrast: F_1, 44.1_ = 0.08, P = 0.780). In 1999, fungicide decreased the number of *P*. *pratensis* seed heads per square meter 44% from 24.7 to 14.0 (1999 fungicide: F_1, 21_ = 7.06, P = 0.015), and decreased the average mass of each *P*. *pratensis* seed head 18% from 0.065 to 0.054 g (1999 fungicide: F_1, 21_ = 4.66, P = 0.043). No other predictor variable or interaction significantly affected *P. pratensis* number of seed heads per square meter or average mass per seed head in 1999.

Fungicide increased the seed mass per square meter of *Carex siccata* by 174% in 1998 (Table 1, Fig. 1D), but had no significant effect in 1999 (1999 fungicide contrast: F_1, 43.8_ = 0.04, P = 0.848). In 1998, fungicide had no significant effect on the number of *C*. *siccata* seed heads per square meter, and increased the average mass of each *C*. *siccata* seed head 53% from 0.011 to 0.016 g (1998 fungicide: F_1, 28_ = 4.79, P = 0.037). No other predictor variable or interaction significantly affected *C*. *siccata* number of seed heads per square meter or average mass per seed head in 1998.

### Number of *A. gerardii* seeds and average seed mass

**Figure 2:**
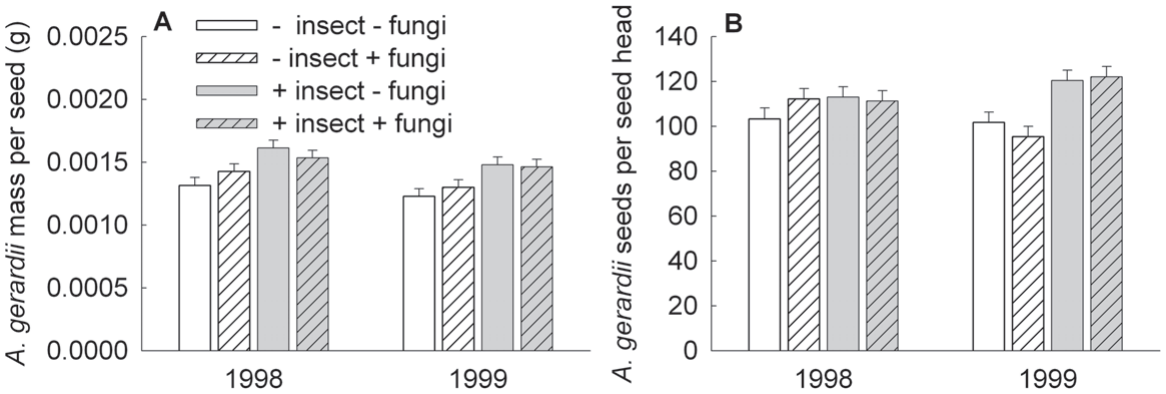
The effects of insecticide, fungicide, and year on additional measures of *Andropogon gerardii* seed production. Effects are shown for (A) mass per seed and (B) seeds per seed head (error bars are +1 standard error).

*Andropogon gerardii* is generally the most dominant plant species in the tallgrass prairie [37], and our treatments affected this species more strongly than the other species across both years. Therefore, we further examined how our treatments affected this species. Insecticide increased the mass per seed of *A*. *gerardii* 16% in both years (Fig. 2A, insecticide: F_1, 28.1_ = 20.31, P<0.001; year: F_1, 28_ = 6.51, P = 0.017). In 1999 insecticide increased the number of *A*. *gerardii* seeds per seed head 23% (Fig. 2B, insecticide x year: F_1, 28_ = 9.40, P = 0.005; insecticide: F_1, 28.2_ = 14.01, P<0.001), but had no significant effect in 1998 (1998 insecticide contrast: F_1, 53.2_ = 0.89, P = 0.351). No other predictor variable or interaction significantly affected *A*. *gerardii* mass per seed or number of seeds per seed head.

### Vegetation biomass and richness

Neither insecticide, fungicide, nor their interaction significantly affected *A*. *gerardii*, *C*. *siccata*, *P*. *pratensis*, or *S*. *scoparium* 1999 aboveground biomass (means = 130.8, 13.1, 51.1, and 18.5 g m^−2^, respectively), or significantly affected plant species richness (mean = 6.4 species per square meter).

## Discussion

Across four plant species in two years, we found no hint of any interactive effects of insect herbivores and fungal pathogens on seed production. It should be noted, however, that the statistical power for each test of an insecticide x fungicide interaction was only 0.277 and 0.589 for “medium” and “large” effect sizes, respectively [36]. The lack of detected interactions was despite strong independent effects of either insect herbivores or fungal pathogens on each species in at least one of the two study years. We suggest that the lack of interactions resulted from complementary resource use by insect herbivores and fungal pathogens in this system, with fungal pathogens directly affecting vegetation and insects directly affecting reproductive structures. All observed fungal pathogens were on leaves, suggesting that pathogens reduced seed production by decreasing plants' resources. Specifically, while pathogens did not decrease aboveground biomass or alter species richness, they decreased plant community root biomass 47%, community leaf longevity 23%, and *A*. *gerardii* photosynthetic capacity 32% [25]. Less than 1% of community leaf area was consumed by insect herbivores [25], suggesting that insects reduced seed production by consuming flowers or seed embryos.

For the more detailed data on *A. gerardii,* the effect in both years of insect exclusion on mass per seed suggests consumption of seed embryos, leaving the seed coat intact. The effect in 1999 on number of seeds per seedhead suggests consumption of flowers. One of the species consuming *A. gerardii* embryos and flowers was likely the Cecidomyiid midge, *Contarinia wattsi* [13]. On the other hand, direct consumption can less easily explain the effect of insect exclusion on the number of seedheads per square meter. Finally, at the plant community level, the species impacted by pathogens were not the same species as were impacted by herbivores. This form of complementarity in resource use will strongly reduce the potential for interactions between pathogens and herbivores. Overall, our results are consistent with the hypothesis that complementary resource use by multiple consumers reduces the potential for interactive effects on host performance [8], [28], [31].

For all plant species studied except for *A. gerardii*, there was an effect of either insect or pathogen exclusion on seed production that varied among years. Interannual variation in the effects of pathogens and insect herbivores on plant performance have commonly been reported in studies that lasted more than one year [10], [18], [26], [28], [34]. We found interesting interannual variation in two years, but even longer studies of grassland seed production would allow a better estimation of how pathogens and insect herbivores affect lifetime plant seed production.

We know of no plausible mechanism by which the fungal exclusion treatment would directly reduce seed production. Therefore, the negative effect of pathogen exclusion on *P. pratensis* seed production in 1999 suggests an indirect effect. Specifically, there may be an indirect effect of fungal pathogens on the competitive dominant *A. gerardii*, and perhaps the less abundant but highly competitive *S. scoparium*. In our experiment, pathogen exclusion increased leaf longevity and photosynthetic capacity of *A. gerardii* [25]. Pathogen exclusion also increased root biomass by nearly 50%. While roots could not be identified to species, *A. gerardii* comprised over half of aboveground biomass and percent cover [25]. Further, *A. gerardii and S. scoparium* allocate more biomass to roots, and reduce soil nitrate to lower levels, than does *P. pratensis* [38]. We hypothesize that pathogen exclusion increased *A. gerardii* root biomass (and perhaps *S. scoparium* root biomass), which reduced soil nitrate availability, resulting in reduced seed production by *P. pratensis* but not reduced vegetative production. This would imply that the negative impacts of pathogens on the competitively dominant species released the subdominant species from competition, resulting in compensatory reproduction. Such compensatory effects have been documented both within host populations [27] and in multi-species communities, in which case it can increase host species diversity [26], [39]. *Poa pratensis* may have been especially vulnerable to root competition due to its relatively high nitrogen requirements [38] and its shallow root system [40]. In contrast, direct negative impacts of fungal pathogens on *C. siccata* appear to have been sufficient to outweigh the indirect benefits of competitive release.

The structure of grassland plant communities may reflect a competition-colonization tradeoff, wherein competitive dominant species are poor colonizers and good colonizers are competitively inferior [6]. In our system, *A. gerardii* has been considered the prime example of the former class of species. Notably, when seed is present, *A. gerardii* establishes and dominates quickly, both on bare ground [41] and in disturbed vegetation [42]. However, *A. gerardii* is consistently slow to colonize successional habitats, suggesting that this is due to lack of long-distance seed dispersal [43], perhaps partially driven by decreased seed production. Thus, the negative impacts of insects on seed production shown here may limit the colonization rate of the competitive dominant species, and thereby contribute to the coexistence and diversity of species in this community.

It is also possible that changes in seed production could cascade to affect organisms in different trophic levels. Specifically, seed availability can affect populations of birds and rodents of conservation concern [44], [45], and a halving of the seed production of *A. gerardii* may strongly affect many granivorous animals. It should also be noted that non-insect granivorous animals were likely affecting the seed production of the four graminoids in this study, even though we did not specifically test for these effects.

Overall, this study shows that insects and fungi can alter seed production for four of the most common graminoids in North America. The dominant species, *A. gerardii*, showed an especially strong and consistent response to insect exclusion. These changes in seed production are likely to have the greatest effects on population dynamics during colonization events. The spread of these dominant graminoids away from established plant populations may be limited by herbivores and pathogens.

## Supporting Information

Dataset S1 (0.08 MB XLS)
